# The use of a policy dialogue to facilitate evidence-informed policy development for improved access to care: the case of the Winnipeg Central Intake Service (WCIS)

**DOI:** 10.1186/s12961-016-0149-5

**Published:** 2016-10-18

**Authors:** Zaheed Damani, Gail MacKean, Eric Bohm, Brie DeMone, Brock Wright, Tom Noseworthy, Jayna Holroyd-Leduc, Deborah A. Marshall

**Affiliations:** 1 Department of Community Health Sciences, Cumming School of Medicine, University of Calgary, 3rd Floor, TRW Building, 3280 Hospital Drive NW, Calgary, AB T2N 4Z6 Canada; 2 Department of Surgery, University of Manitoba, AE101 – 820 Sherbrook Street, Winnipeg, MB R3T 2N2 Canada; 3 Manitoba Health, Healthy Living and Seniors, Acute, Tertiary and Specialty Care/Regional Policy and Programs, 2061- 300 Carlton Street, Winnipeg, MB R3B 3M9 Canada; 4 Winnipeg Regional Health Authority, 4th Floor - 650 Main Street, Winnipeg, MB R3B 1E2 Canada; 5 Department of Community Health Sciences, University of Calgary, Cumming School of Medicine, 3C56, Health Research Innovation Center (HRIC) Building, 3280 Hospital Drive NW, Calgary, AB T2N 4Z6 Canada

**Keywords:** Deliberative dialogue, Health policy, Research evidence, Knowledge translation, Public participation, Healthcare decision making, Evidence-informed decision making, Health systems, Access, Surgical procedures

## Abstract

**Background:**

Policy dialogues are critical for developing responsive, effective, sustainable, evidence-informed policy. Our multidisciplinary team, including researchers, physicians and senior decision-makers, comprehensively evaluated The Winnipeg Central Intake Service, a single-entry model in Winnipeg, Manitoba, to improve patient access to hip/knee replacement surgery. We used the evaluation findings to develop five evidence-informed policy directions to help improve access to scheduled clinical services across Manitoba. Using guiding principles of public participation processes, we hosted a policy roundtable meeting to engage stakeholders and use their input to refine the policy directions. Here, we report on the use and input of a policy roundtable meeting and its role in contributing to the development of evidence-informed policy.

**Methods:**

Our evidence-informed policy directions focused on formal measurement/monitoring of quality, central intake as a preferred model for service delivery, provincial scope, transparent processes/performance indicators, and patient choice of provider. We held a policy roundtable meeting and used outcomes of facilitated discussions to refine these directions. Individuals from our team and six stakeholder groups across Manitoba participated (*n* = 44), including patients, family physicians, orthopaedic surgeons, surgical office assistants, Winnipeg Central Intake team, and administrators/managers. We developed evaluation forms to assess the meeting process, and collected decision-maker partners’ perspectives on the value of the policy roundtable meeting and use of policy directions to improve access to scheduled clinical services after the meeting, and again 15 months later. We analyzed roundtable and evaluation data using thematic analysis to identify key themes.

**Results:**

Four key findings emerged. First, participants supported all policy directions, with revisions and key implementation considerations identified. Second, participants felt the policy roundtable meeting achieved its purpose (to engage stakeholders, elicit feedback, refine policy directions). Third, our decision-maker partners’ expectations of the policy roundtable meeting were exceeded; they re-affirmed its value and described the refined policy directions as foundational to establishing the vocabulary, vision and framework for improving access to scheduled clinical services in Manitoba. Finally, our adaptation of key design elements was conducive to discussion of issues surrounding access to care.

**Conclusions:**

Our policy roundtable process was an effective tool for acquiring broad input from stakeholders, refining policy directions and forming the necessary consensus starting points to move towards evidence-informed policy.

## Background

Responsive, effective, evidence-informed policy has been lacking in healthcare. This has prompted debates around how best to engage, develop and implement such policy [1], leading to a focus around greater public participation and stakeholder engagement. Traditional models of policy development have been centred on interests, ideas and institutions, or problems, policies (or solutions) and politics (or political events) as key forces in shaping policy outcomes, policy directions or agenda setting, each flowing in independent streams [2, 3]. Such notions and models of policy development are increasingly seen as simplistic and no longer reflective of current decision-making processes and no longer appropriate for “*a more educated, sophisticated and less deferential public*” [1, 2, 4, 5].

### Need for more deliberation and broader participation in policy development

Concerted efforts for broader and increased engagement include more deliberate involvement of both “insiders” (participants with close proximity/direct involvement) and “outsiders” (those with less proximity/direct involvement) and the use of innovative tools and stronger emphasis on implementation. Together, these are leading to more vehicles for engagement. Working definitions, for the purpose of this manuscript, are presented below. A roundtable meeting, in its simplest form, is meant to provide a forum for discussion and debate around a specific topic. Engagement processes enable the coming together of individuals and organisations for democratic and inclusive decision-making. These differ from deliberative processes, which allow “*a group of actors to receive and exchange information, to critically examine an issue and to arrive at an agreement that informs decision-making*” [6], and deliberative dialogues, which are “*a type of group process that can help to integrate and interpret scientific and contextual evidence for the purpose of informing policy development*” [7]. These dialogues use research evidence to help address challenges faced by both policymakers and stakeholders [8]. Finally, “policy dialogues” can be seen as a form of deliberative dialogue, described as “*component*[s] *of the policy and decision-making process… intended to contribute to informing, developing or implementing a policy change following a round of evidence-based discussions, workshops, and consultations on a particular subject*” [9].

Central to any collaborative engagement and deliberation is the alignment of “*beliefs, values, interests and goals/strategies of elected officials and social interest groups*” [2, 10, 11]. Having multidisciplinary teams and decision-makers working alongside engaged researchers is seen as critical [12, 13] for the uptake of research evidence [8, 10, 11, 14–16] and its translation into evidence-informed policies for improved quality of care and strengthened health systems. The growing number of stakeholders in each category of “producer” and “user” of knowledge necessitates multiple mechanisms that can allow the voices and perspectives of all to be considered if outcomes of their interactions (i.e. policy) are to be innovative, inclusive and responsive at both the levels of production and use. These mechanisms are increasingly being used to develop responsive, inclusive, effective and evidence-informed policy. While there is emerging evidence to support the use of deliberative mechanisms, such as deliberative dialogues, deliberative processes and policy dialogues, evaluations of their effectiveness are lacking, especially in health [6–8].

### Access to care: more attention and involvement needed

Access to care remains chronically problematic in Canada, especially for scheduled clinical services such as total hip and knee joint replacement (TJR) [17–19]. Stagnating waiting times internationally have prompted calls for innovative models of care and pressure on policy and decision-makers for sustainable, effective solutions. As systems become burdened by increases in the aging population, disease prevalence and costs in the face of limited resources and competing priorities, vehicles for stakeholder engagement can be important contributions to evidence-informed policymaking and represent a special opportunity to develop collaborative solutions that span the continuum of care and reflect the perspectives and priorities of those that will both contribute to and benefit from them [6, 14, 20, 21].

### Single-entry models (SEMs): a unique tool for access improvement requiring more evaluation

SEMs have been suggested by the Canadian Institutes for Health Information and the Organisation for Economic Co-operation and Development as a promising waiting time management strategy that brings structural and programmatic improvements to the management of patients awaiting elective procedures [22–24]. In healthcare, they often combine the use of pooled lists (patients and service providers), centralised intake (single point-of-entry) and triage (screening for urgency/priority) to improve the management of referrals and patients awaiting access to scheduled services. The use and comprehensive evaluation of these models in Canada is limited. In 2012, the Winnipeg Regional Health Authority (WRHA) implemented the Winnipeg Central Intake Service (WCIS), a SEM to improve patient access to TJR in Winnipeg, Manitoba. Our multidisciplinary team, including researchers, administrators and physicians, partnered with senior decision-makers from the Government of Manitoba (Manitoba Health, Healthy Living and Seniors) and the WRHA to comprehensively evaluate the WCIS. We used our findings to develop evidence-informed policy directions and hosted a policy roundtable meeting to bring together stakeholders to critically examine and refine them, in order to help improve access to scheduled clinical services across Manitoba. We report here on the policy roundtable process and its role in contributing to the development of evidence-informed policy.

## Methods

### Development of policy directions

The comprehensive evaluation of the WCIS – its planning, implementation and resulting outcomes – took place from March 2013 to July 2014. We employed a mixed-methods case study approach (qualitative interviews and administrative data analysis) to examine the implementation process and to determine the influence of SEMs on health and health service delivery. The quality of health service delivery was assessed using six dimensions of quality of care: Acceptability, Accessibility, Appropriateness, Effectiveness, Efficiency, and Safety. A total of 131 semi-structured interviews were conducted with individuals from five stakeholder groups involved in or affected by the WCIS, namely patients, family physicians, orthopaedic surgeons, surgical office assistants, and WCIS team members (including decision-makers), during and after the implementation of the WCIS. Stakeholders were asked about their experiences with the WCIS, specifically what worked well, what did not, and ways in which both the WCIS and their experiences could be improved. Two coders synthesised the data using thematic analysis to identify, classify and organise key themes, major findings and stakeholder recommendations (described elsewhere [25–29]). The research team and decision-maker partners used these findings to co-develop five related evidence-informed policy directions during an in-person meeting, not only to improve the implementation of the SEM for TJR, but also to broadly improve access to scheduled clinical services across Manitoba (Box 1).

**Box 1:** Five policy directions (with overview) that were presented for discussion at our policy roundtable meeting

1. *The quality of health service delivery should be measured and monitored according to a provincial framework* This was presented as the first policy direction as it is foundational for the successful establishment of a single-entry model (SEM). The impetus for the establishment and success of the Winnipeg Central Intake Service stems from the grounding of its processes in data, enabling the rigorous tracking and monitoring of patient referrals and data related to health service delivery and outcomes. Along with established guidelines for data collection, it is necessary for data to be monitored according to agreed-upon criteria that would be consistent across the province.
2. *Central intake should be the preferred model for service delivery of scheduled clinical services* With considerations for data collection, management and privacy in place, SEMs represent a strong alternative to traditional models of patient management. SEMs are especially well suited to choice-sensitive, elective scheduled procedures. This policy direction served to position centralised intake as the preferred model for management of patients awaiting scheduled services.
3. *Central intake programs for scheduled clinical services should be provincial, where appropriate* With common guidelines and processes in place, resources can more efficiently be shared across the province – whether personnel capacity, operating room time or other resources – for both patient and system-level benefits. This can also allow for access based on patient preference and proximity, while harnessing provincial capacity to the fullest extent possible.
4. *Central intake structure and processes, as well as relevant performance indicators of patient and system outcomes should be made available in a transparent fashion to the public and health providers* Patients feel more at ease and less anxious when more fully informed about what can and should be anticipated as they prepare for their procedure – whether this includes the anticipated date of surgery or breadth of education classes available. As part of efforts to increase accountability, patient communication and shared decision-making, data that is being collected can and should be shared to increase patient and provider confidence in the efficacy and safety of SEMs. This reporting can also be used to strengthen health system performance and inform governance and future planning.
5. *Patients should maintain the choice of seeing the first-available specialist or specialist of their choice for a scheduled service* In addition to more efficient management and expeditious access, at their core, SEMs are meant to increase rather than decrease patient choice. The hallmark of the success of SEMs is their ability to both respect patient choice – to see either a specific or next-available surgeon – while improving access.

### The policy roundtable meeting

We hosted a policy roundtable meeting that brought together individuals from a range of stakeholder groups across Manitoba, including patients. The policy roundtable meeting objectives and format were developed by the research team and policymaker partners, using key elements and guiding principles of policy dialogues suggested by the Canadian Institutes of Health Research, The National Collaborating Centre for Healthy Public Policy and the Canadian Health Services Research Foundation (now Canadian Foundation for Healthcare Improvement; see Table 1 for all design features of the meeting) [30–32]. The roundtable meeting had the following format: (1) addresses a high-priority issue; (2) clear objectives; (3) an environment conducive for deliberations; (4) clear rules of engagement; (5) consultation of those who will be affected by the issue/outcome; (6) a mix of participants/stakeholders that represents all relevant perspectives and interests (including representation of researchers); (7) a synthesis of high-quality research evidence and its use to identify needs and to adequately inform/educate participants; (8) opportunities for discussion; (9) not emphasising the need for consensus among participants; (10) skilled facilitation; (11) outcome evaluation; and (12) outputs produced and follow-up activities undertaken [6–8, 30, 33, 34]. This format allowed for stakeholders bringing a diversity of perspectives to discuss and refine the policy directions.

**Table 1:** Comparing key elements and guiding principles of “deliberative dialogues” to the design of our policy roundtable meeting

Element [6–8, 30, 33, 34]	Present	Elements/details – policy roundtable meeting
Addresses a high-priority issue	Yes	• Of national and international concern; improving access to elective total joint replacement surgery of the hip and knee in Manitoba, Canada
Clear meeting objectives	Yes	• Clear objectives, articulated in advance
Pre-circulated information package	No	• Participants were provided with nametags and folders upon their arrival containing an agenda, list of policy directions to be discussed, related background information and an evaluation form; table seating was pre-assigned (eight participants (mixed backgrounds) per table with one recorder, one facilitator)
Pre-circulated evidence summaries	No	• To avoid social desirability bias during discussions, where possible, and to elicit the most authentic reactions/responses from participants based on their experience and knowledge; to best identify where/how participant views converge
Environment conducive for deliberations	Yes	• Downtown hotel ballroom (central location); presentation-style room set-up with round tables, flip charts, easels, post-it notes and pens at each table • Frequent breaks, meals and honoraria provided • Meeting was scheduled from 10:00 am – 3:00 pm, to allow for participant travel, maximise productivity and to reduce fatigue
Clear rules of engagement/task definition	Yes	• Overview provided by both facilitators and decision-maker research partners (WRHA and Manitoba Health) to set the tone and establish a safe, inclusive, non-judgmental and respectful space for discussion • Nature and scope of the meeting and exercise was clearly defined
Recording of discussions	Yes	• Discussions related to the policy directions
Consultation of those who will be affected by issues	Yes	• Meeting was attended by five stakeholder groups, with participants attending from across Manitoba
Mix of participants and stakeholders representing all perspectives and interests	Yes	• Participants purposively selected to contribute to the policy discussion • Assigned small group seating to maximise variation of perspectives at each table
Representation of researchers and decision-makers	Yes	• Meeting prior to the commencement of the policy roundtable meeting to ensure comfort, alignment and understanding of objectives, agenda • Identified to all participants • Presented during opening sessions • Played the role of discussion facilitators and “recorders” at each table • Presented summaries and next-steps at the conclusion of the meeting
Synthesis of high-quality research evidence used to identify needs and educate participants	Yes	• Synthesis of research, findings in the form of four short, pre-discussion presentations by research team members: – Pertinent background information on waiting times in Canada, issues of access and the concept of ‘queuing’ and centralised intake – Pertinent background information on single-entry models as an evidence-informed strategy to address waiting times and the local context related to hip and knee replacement surgery in Winnipeg, leading to the development of the WCIS (i.e. the problem to be addressed); – Results of the research team’s comprehensive evaluation of the WCIS – sharing of perspectives from all five stakeholders – Formal Introduction and Welcome by decision-maker research partners; setting the tone and providing an overview of the policy directions for discussion • Results of research team’s evaluation were used to inform development of carefully considered evidence-informed policy directions • Policy directions were the focus of the afternoon discussion/small-group sessions
Opportunities for discussion	Yes	• Opportunities provided to discuss the problem, possible solutions/approaches and considerations for ameliorated implementation through breakout sessions and open dialogue
Not emphasising need for consensus	Yes	• Casual, collegial atmosphere, with a focus on the need to work collaboratively and for all voices to be heard, perspectives to be shared • Questions developed to focus discussion on the policy directions • Table facilitators to support a more equal ‘playing field’ • Recorders at each table to capture details of discussion
Skilled facilitation	Yes	• External, respected facilitator from outside of the research team • Welcomed participants; began with introduction to set the tone, expectations and to establish comfort and a safe, non-judgmental space • Ensured breakout sessions for policy direction discussion were kept to 45 minutes per policy direction – policy directions 1 and 2 were discussed, followed by lunch, and then 3–5 • Facilitator moderated the (1) sharing of feedback and results from each group during the report-back session, (2) open discussion and (3) summary and next steps/closing
Outcome evaluation	Yes	• Post-meeting evaluation forms
Outputs produced, follow-up activities undertaken	Yes	• Follow-up national-level Policy Roundtable Meeting: Canadian Symposium on Single-Entry Models, hosted in Ottawa, Canada in April 2015 • Follow-up with our policy/decision-maker partners after the policy roundtable meeting and again 15 months following the policy roundtable meeting for their perspectives on the value of the meeting and for updates on the impact and use of the policy directions to improve access to scheduled clinical services in Manitoba

Policy dialogues generally involve discussion about multiple aspects of a problem, possible approaches to addressing the problem, and considerations for implementation [33]. Our policy roundtable meeting was purposively structured to address these three issues. The objectives of this policy roundtable meeting were (1) to share and discuss policy directions with respect to the implementation of central intake models for scheduled clinical services across Manitoba; (2) refine them based on feedback from stakeholder consultation; and (3) to assess the policy roundtable process and its early impact.

Invitations were extended to 50 individuals identified by the researchers and decision-maker partners. A total of 44 individuals attended from across Manitoba and brought with them a variety of experiences and perspectives. Twenty-two participants attended from the five stakeholder groups that were targeted for our WCIS qualitative evaluation (patients (*n* = 4), family physicians (*n* = 2), orthopaedic surgeons (*n* = 1), surgical office assistants (*n* = 2), the WCIS team members (*n* = 13)) and members of our national research team (*n* = 10). These participants brought with them experience with the WCIS for TJR. Also participating were medical and administrative leaders invited as stakeholders from other health regions and health organisations in Manitoba (*n* = 12).

This full-day meeting was held in Winnipeg, Manitoba, on June 9, 2014, and was led by an independent facilitator who was neither a member of the research team nor a stakeholder. Along with the facilitator, the team sought to create an inclusive, safe environment conducive to open, non-judgmental discussion and dialogue.

The meeting commenced with presentations by members of the research team to introduce context and background information related to (1) the issues of access to TJR in Manitoba and Winnipeg in particular; (2) design of the WCIS and use of SEMs as a way to increase capacity, variability, flow and, consequently, waiting times; (3) research evidence (results from the evaluation of the WCIS); and (4) the five proposed evidence-informed policy directions for consideration of the meeting participants, which, if pursued and implemented, could help improve access to scheduled clinical services across Manitoba. All invited participants were asked to reflect on the five proposed policy directions, to discuss their relevance, feasibility and clarity, to consider implementation issues, and to discuss the potential implications for health and health system improvement. They were framed as high-level policy directions for discussion, looking at the potential use of central intake models across the province for scheduled clinical services including, but beyond, TJR.

In-depth discussion of these five policy directions took place in small groups, with the conversation facilitated. Seating arrangements had been pre-determined to ensure a mix of participation at each of the five tables. Facilitators tried to create an environment where all participants felt comfortable speaking, so that each participant’s perspectives could be obtained. Participants were asked to focus on broader policy directions (versus issues that may be operational in nature) and to think about the applicability of the directions to their own regions and to think beyond orthopaedics with respect to scalability and benefit for others looking to implement a central intake service. Following the small group deliberations, the lead facilitator engaged all participants in a large group discussion that allowed for all groups and stakeholders to highlight the key points emerging from their conversations, and to say whether their group agreed in principle with the policy direction, felt that the policy directions should be pursued, and identify which policy directions were of the highest priority.

### Data collection and analysis

Data were collected from the small group discussions by assigned recorders, who carefully documented conversations being guided by assigned facilitators from the research team and policymaker partners. Stakeholders were asked for advice on how to refine, clarify and/or tailor the policy directions for their particular policy environment (i.e. for family members, for the Province of Manitoba, etc.) [32]. Guiding questions were co-developed and refined iteratively by the research team and policymaker partners. Two overarching questions were developed to help reflect stakeholders’ consideration of both the evidence presented and their own personal knowledge and experiences: (1) Do you agree in principle with the policy direction? (2) Should the policy directions be pursued? Five additional questions were developed and used to facilitate discussion on each of the policy directions: What are the benefits with this policy direction? What are the concerns or issues with this policy direction? What are the additional considerations of proposing such a policy direction? How can we ameliorate these concerns and implement this policy direction? Any other suggestions?

The large group discussion was also captured by recorders. All collected data were collated at the end of the day and imported into a single Microsoft Word document. Two independent raters coded the data and used thematic analysis to identify concepts, and organise relationships between concepts raised at all discussion tables [35], under each of the five policy directions that could be used to help further refine them. Agreement on themes was reached by consensus.

A post-meeting evaluation instrument was developed to obtain participants’ assessment of the policy roundtable. It included 20 items divided into six sections (to reflect some of the key design elements of participatory and deliberative processes, with blank fields for comments, where relevant), and was completed by participants immediately at the conclusion of the roundtable meeting. The first section evaluated the extent to which the meeting objectives were met and purpose fulfilled, including value of attending, and whether policy directions reflect the relevant policy considerations for centralised intake. Additional sections inquired about participants’ meeting experience and opportunity to hear and be heard during activities. Finally, open-ended questions were included for participants to share comments or suggestions they were unable to share during the meeting.

Data were entered into and analysed using Microsoft Excel, and summarised using basic descriptive statistics. Written comments and responses to the open-ended questions were analysed using thematic analysis to identify, organise and categorise emerging themes. All completed questionnaires were anonymous and confidentially completed, and stored in a locked filing cabinet.

Immediately following the policy roundtable meeting, members of the research team conducted an informal interview with each of the policy/decision-maker partners from WRHA and Manitoba Health, Healthy Living, and Seniors (*n* = 3) to obtain their perspectives on the policy roundtable meeting, including its value and impact. Events of the meeting were discussed, including differences between expectations and outcomes. Policy/decision-maker partners were contacted again 15 months following the policy roundtable meeting to share their perspectives and updates on the impact and use of the policy directions to improve access to scheduled clinical services in Manitoba. The data collected through these discussions were analysed using thematic analysis.

## Results

### Participant assessment of the policy directions

Participants showed great enthusiasm during and after the group discussions and final session dedicated to sharing key ideas. They agreed that all five of the proposed policy directions were critical for the improvement of access to scheduled clinical services. Participants generally shared common areas of agreement and disagreement, which allowed for the policy directions to be prioritised accordingly. Specifically, policy directions #1 (measurement and monitoring) and #5 (patient-centred approach, with patients retaining choice of provider) were seen as instrumental to quality assurance and particularly critical for any further improvement in Manitoba. Additionally, participants agreed that central intake should be the preferred model for service delivery (policy direction #2) and on a provincial scale (policy direction #3), but only where well suited (choice-sensitive, elective procedures for which waiting time is variable). Finally, participants agreed that mechanisms must be in place for quality assurance and that structure, processes and performance reporting should be public (policy direction #4), but with careful thought given as to what is reported and how.

### Refining policy directions

An overarching provincial standards committee, to ensure that anyone who participates in a central intake process can expect to receive high quality care, was suggested as one mechanism for this.

Through the roundtable discussions, increased understanding and communication stood out as core for how one might report on quality in a way that is meaningful to patients, families and practitioners. Patients shared that they were less concerned about specific quality metrics. They preferred knowing that (1) a process exists to monitor quality and outcomes and to be able to trust that the surgeon to whom they are being referred does high quality work and that (2) the difference in likely waiting times between choosing a particular surgeon and choosing the first-available surgeon. Patients emphasised that both are required to make an informed choice, something they often do in collaboration with their family physician. Key ideas, themes and implementation considerations from participant small group discussions of each policy direction are outlined in Table 2. Summaries of the large group discussion are highlighted in Box 2.

**Table 2:** Stakeholder perspectives and responses to policy direction discussion questions (breakout discussion groups)

Questions	Policy Direction #1: Measurement and monitoring according to a provincial framework	Policy Direction #2: Central intake as preferred model for delivery of services	Policy Direction #3: Central intake programs should be provincial	Policy Direction #4: Central intake structure, processes, performance should be public	Policy Direction #5: Patients should maintain the choice of who they see
Do you agree in principle with the policy direction?	- Yes	- Yes, but not universally; single-entry models (SEMs) may not be well suited to all clinical services	- Yes	- Yes	- Yes
Should it be pursued?	- Yes. Seen as “*foundational*” to the other policy directions	- Yes, where well suited	- Yes, but it must be acknowledged that it may not be suited to all clinical areas	- Yes, but careful thought must be given as to what should be reported and how well it could be understood	- Yes
Benefits	- Measurement, monitoring and accountability framework - Would lead to benchmarks with actionable data and reporting - Would establish transparency, standards to which processes could adhere, regardless of jurisdiction	- SEMs are well suited to choice-sensitive elective procedures, especially those with high volumes, long waits and variabilities - Central intake creates a platform for starting to have clinical service agreements between primary and secondary care - Potential for improved quality of care, governance and oversight	- Can facilitate improved access, sharing of resources - Enables a provincial mechanism for quality assurance in all areas where care is being delivered	- Better information, reporting and decision-making - Patients/members of public have right to know information - Increased transparency could lead to improved accountability and encourage continual improvement - Assurance to patients that processes are in place to ensure adherence to standards of quality of care	- A patient-centred approach - Patient confidence - More patient control - Helps establish/maintain relationships between providers
Concerns	- May lead to unknown opportunity costs, unintended consequences, gaming - Will not be effective unless participation is 100% - Results of monitoring will be highly context dependent	- Cannot and should not be applied universally - Potential depersonalisation, reduced accountability and reduced appropriateness of referrals	- May be challenging to gain consensus on a provincial quality metric that can be collected and used across regions - Rural patients may be at a disadvantage - Rural providers – fear of loss of patients to larger centres - Management/coordination	- Data may not be valued or understood - Too much information can be overwhelming and/or misunderstood - Implications of the reporting could lead to gaming or skewed expectations from public	- Referring physicians may not know all of the options available - General public does not fully understand how care is provided/referrals are directed - Patients can sometimes have unrealistic expectations - Acceptance will never be universal
Additional considerations of proposing this policy direction	- Must be a coherent effort, and not completed in isolation; apply to all - There will need to be a standardised approach – consensus and agreement around which indicators should be measured - Measurement and monitoring should be established on a provincial basis, not only regionally	- Standardisation of processes, quality measures, referral criteria, feedback mechanism - Data-driven process (to help measure demand/patient volume); data could facilitate non-threatening dialogue - Patients able to retain choice - Incremental implementation	- Must have clear purpose/processes/provincial standards for quality – ensures transparency and flow of resources across regions - There should be a focus on equity for all - Patients must be able to retain choice - Central intake does not mean central provision - How will resources be distributed to meet demand – by volume? Per capita?	- Involvement of all stakeholders will be important for shared buy-in and use - Incorporation of patient-reported outcome measures - Patients should be able to retain choice - Smaller selection of measures may be more meaningful - Standards, processes, purpose of this data and implications should be clearly communicated to relevant stakeholders	- Information sharing will be required so that patients and referring physicians can be informed and help patients make the best decision - With measuring and monitoring in place, confidence can be increased in providers across the system so that patients can feel more confident - Feedback mechanisms will be needed for continual improvement - Quality of care should not differ for patients who see the next-available vs. specific surgeon - Patients will need to understand that their choice may involve a longer wait
How can we ameliorate these concerns and implement this policy direction?	- Begin with small, defined first steps and expand over time as capacity develops more fully	- Where promising, the scope for SEMs should be well studied to ensure the context/environment is conducive to success - Care could be elevated to a system level, where all providers working together for the best care possible - Strong infrastructure needed	- Consideration should be paid to ensure that any care pathway is not burdensome to patients - Incremental implementation	- Aggregate reporting may be best	- As patients and providers are well informed and get used to the system, trust of the system and processes will increase
Additional comments	“*We cannot improve if you don’t know how you’re doing*”

**Box 2:** Policy direction participant group discussion summary

*Policy Direction #1: The quality of health service delivered should be measured and monitored according to a provincial framework* There was enthusiasm and a consensus feeling among attendees that the concept of ‘measurement’ and ‘monitoring’ is critical to moving forward with a centralised intake and that this particular policy direction is foundational to the others.
*Policy Direction #2: Central intake should be the preferred model for service delivery of scheduled clinical services* Participants agreed that, while suited to choice-sensitive elective procedures, single-entry models are not universally applicable, needed or possible across all clinical services in all regions/jurisdictions.
*Policy Direction #3: Central intake programs for scheduled clinical services should be provincial, where appropriate* There was a realisation that centralised intake is part of a larger system, part of a continuum, but does not constitute the whole system. It would, however, serve as an opportunity for system level improvements, whereby it results in providers working collaboratively to provide quality care.
*Policy Direction #4: Processes for central intake and performance indicators for patient and system outcomes should be made transparent to the public and to providers* There was agreement that a provincial single-entry model can facilitate improved access (via better tracking, measuring and monitoring) and better sharing of resources for everybody in the province. Where in use, a provincial model should be governed by provincial standards for quality (to ensure transparency) and allow resources to flow across regions. Careful thought needs to be given as to what should be reported and how well it could be understood. Increased transparency could mean increased accountability and put pressure on the system for continual improvement. Such reporting can also provide assurance to patients that processes are in place to ensure high quality of care standards.
*Policy Direction #5: Patients should maintain the choice of seeing the first-available specialist or specialist of their choice for a scheduled service* There was strong consensus that patients must be able to maintain their choice. Patients have varying degrees to which they seek advice and support in their decision-making. With measuring and monitoring in place, confidence can be increased in providers across the system so that patients can feel more confident. Patient choice is important to retain with the introduction of any central intake process for scheduled and elected surgery. Quality of care should not differ for patients who choose to see a next-available versus a specific surgeon.

### Assessing the policy roundtable meeting process

The majority of the invited participants completed the policy roundtable meeting evaluation questionnaire (*n* = 31/34), with a response rate of 91%. The nine members of the research team and the meeting facilitator participating in the meeting did not complete the questionnaire.

Feedback was generally positive with more than 96% of respondents rating 18 out of 19 questions as ‘agreed’ or ‘strongly agreed’ (Table 3). Feedback showed that participants were engaged and felt like they were able to contribute meaningfully. It also shows that the design elements of our meeting (14/16 design elements of best practice were met) made for a productive meeting that fulfilled the goals, provided an environment conducive to dialogue, effectively elicited participant response, and yielded the desired and intended effects of our deliberative mechanism. Areas with the most positive feedback were related to meeting objectives being met, and the relevance of the discussion.

**Table 3:** Results of participant evaluations (response rate: 91% (31/34))

Question	Agreed or Strongly agreed (*n* = 26–31)
Background knowledge/presentations	
Presentations provided me with the background knowledge and understanding to contribute to the meeting	90%
Meeting objectives	
Meeting objectives were met	97%
I understood the material presented	94%
The meeting was valuable to attend	93%
My voice was heard when discussing the policy directions	87%
The policy directions presented reflect the relevant policy considerations for centralised intake	97%
Meeting experience	
The meeting brought together relevant stakeholders	97%
I had the opportunity to share ideas and issues	93%
The meeting was relevant to me or my work	97%
I made at least one new contact	90%
I plan to act or share learning from the meeting with a colleague not involved with the meeting	83%

### Post-meeting data collection

The decision-maker members of the team described the process as very valuable and “*exceeding their expectations*”. It provided them an opportunity to bring together regions across the province that were not using a central intake process, to share some of the research findings and then engage stakeholders in a larger discussion regarding how the province might approach access to scheduled clinical services in the future. They felt the meeting (1) was a good starting point to engage people across the province in discussing central intake through SEMs and their elements (i.e. pooled lists, centralised intake, triage) as a way for improving access by decreasing the variability and length of waiting times; (2) provided a forum to bring together a variety of diverse stakeholders for an in-depth, bigger picture consideration of issues; (3) provided an opportunity to test and observe stakeholder receptivity towards SEMs, and related policy implications. One of the senior decision-makers remarked: “*This kind of discussion would not have been possible 10 years ago*”. Collectively, they described the stakeholder participation and dialogue (especially patients) as beneficial and observed that there was far more acceptance of and agreement about the five policy directions than was expected going into the policy roundtable meeting. The senior decision-makers on the research team were very supportive of the policy directions and roundtable meeting, in part because they contributed to their development.

These decision-maker partners were contacted 15 months following the policy roundtable meeting and asked about updates and perspectives towards the policy directions. They reiterated their initial perspectives on the value of the policy roundtable, saying that the meeting had gone well and that their expectations had been exceeded, citing the following as positive attributes: good discussion and thorough consideration of the issues, open dialogue and the creation of momentum for SEMs. Some of the major benefits of the meeting included (1) overall increased engagement of stakeholders (including of patients, and others from across the continuum of care); (2) increased understanding and awareness of central intake models and their implications among stakeholders and across Manitoba; (3) a change in mindsets towards the use of central intake processes, and more receptivity towards a wider provincial model applicable to a variety of scheduled clinical services; (4) agreement among major Manitoba stakeholders regarding the principles underlying central intake (SEMs) for scheduled clinical services; and (5) the need for common parameters (ensuring quality, transparency, choice) and a common language around which central intake processes can be developed. Furthermore, the principles and refined policy directions resulting from the roundtable meeting were described as foundational to the vocabulary, vision and framework that had been established following the policy roundtable meeting, and being actively used to help improve access to scheduled clinical services in Manitoba through new provincial planning processes focused on developing a more sustainable heath service delivery model. They described that a provincial orthopaedic standards and quality committee is being developed and will continue to be informed by results from our roundtable meeting to provide a quality assurance mechanism for the WCIS and subsequent central intake processes for improved access to scheduled clinical services across Manitoba.

Decision-makers reiterated that the principles defined in the policy roundtable are the principles that will underlie the resulting central intake processes, namely (1) measurement/monitoring of quality; (2) central intake as a preferred model for service delivery; (3) provincial scope; (4) transparent processes/performance indicators; and (5) patient choice of provider. When asked what could have been done differently, they cited more actionable items/impetus for action and development of clearer next steps that could be followed.

## Discussion

Four key findings emerged from the policy roundtable process and evaluation. First, participant engagement and agreement on nearly every policy direction discussed suggests that the policy directions resonated with the stakeholders and that their consideration will be critical for the improvement of access to scheduled clinical services in Manitoba. Second, participants rated their experiences favourably, suggesting that participants feel that the policy roundtable meeting achieved its purpose (to engage stakeholders, elicit feedback, refine policy directions) and was useful. Thirdly, that our decision-maker partners’ expectations of the policy roundtable had been exceeded and that they were able to be part of work they felt “*would not have been possible 10 years ago*” speaks to the importance of aligned objectives, receptivity towards and effectiveness of researcher involvement, use of research evidence, co-development of evidence-informed policy directions, increased participation and engagement of stakeholders for diverse perspectives to help refine and gain consensus around these policy directions, and use of collaborative processes to help develop inclusive, evidence-informed policy to improve access to care. Finally, the success of the policy roundtable meeting suggests that our tailored approach and adaptation of the key design elements for deliberative processes worked well for the discussion of evidence-informed policy directions for improving access to scheduled clinical services.

Both the WCIS evaluation and the policy roundtable meeting were a collaborative undertaking and an example of bi-directional, integrated knowledge translation and exchange involving decision-maker partners from the start. Regular meetings with the entire research team ensured that the policy questions drove the research questions initially, and that the development of the policy directions discussed at the policy roundtable meeting were based on the research findings. As a result of the meeting, the refined proposed evidence-informed policy directions are more responsive, inclusive and better developed, enabling our decision-maker partners to better identify, assess and respond to patient and system needs and policy objectives more effectively.

### Implications for improved access and SEMs in Manitoba

Decision-maker perspectives following the roundtable meeting demonstrate some of the “*intended effects of deliberative processes*” as a knowledge translation strategy, such as strengthened “*personal capacity to address the policy issue*” (short-term individual-level intended effect). Decision-maker partners also demonstrated dedication and commitment towards strengthening capacity to participate in the agenda setting process or to “*take action if a policy window opens*” (medium-term organisational-level intended effect) and strengthening system capacity to make evidence-informed decisions (long-term system-level intended effect) [8]. Decision-makers also expressed the need for continued engagement and efforts towards fully realising the benefits, enthusiasm and move towards consensus generated by the meeting. This could be accomplished through ongoing teamwork, clear plans and accountability for next-steps, especially when all stakeholders seem to have buy-in and commitment.

### What does this study add to the knowledge base?

Our policy roundtable meeting is among the only ones to be reported and evaluated in the realm of access to care/scheduled clinical services. The Canadian Medical Association hosted a similar meeting to discuss streamlined referral processes in December 2011 [36]. Such processes can facilitate the development of more responsive, effective, evidence-informed policy by reflecting a diversity of perspectives and serving as critical tools for knowledge translation and exchange for the uptake of research evidence by decision-makers [1, 2, 8, 14, 30, 31, 33]. They have been employed in many spheres of human endeavour, and in many countries around the world successfully [1, 2, 14, 30, 37], but their formal description and evaluation have been lacking. Our paper is among the first to describe and assess such a process and its early impact related to the improvement of access to care.

### Limitations

We obtained feedback from participants through the use of a short questionnaire. It may have been useful to also do key informant interviews with a purposive sample of the participants, perhaps 1–2 weeks following the policy roundtable meeting, in order to triangulate these findings. While successful, our policy roundtable meeting could have been more rigorous had it adhered to additional design features, which would have elevated it to a true “*deliberative dialogue*” process [8]. These include circulation of materials in advance, more rigorous outcome evaluations, the production of better outputs for dissemination and more defined short-/medium-term follow-up activities (i.e. for validation by participants) [6, 8, 30, 33].

### Future directions

Further evaluation of deliberative mechanisms can help distil the elements required to better empower stakeholder groups and encourage wider participation, with both process and outcome evaluations required to generate valuable knowledge. Longer-term outcome evaluations are needed to monitor change in intended outcomes; that is, determine whether or not the process was “*effective in causing the intended changes*” [6]. A challenge with outcome evaluations, however, given that the desired change is a change in policy and/or practice, is attribution given the many contributing factors to desired evidence-informed change. Finally, longer-term evaluations are also needed to identify any positive and negative intended and unintended consequences of deliberative mechanisms.

## Conclusions

Our policy roundtable process provided a versatile vehicle to facilitate critical consideration of evidence and context, rich discussion and refinement of policy directions. Acquiring broad input from a diversity of participating stakeholders helped form the necessary consensus starting points to move towards evidence-informed policy that is likely to be more responsive, implementable and sustainable while providing insight and direction on how future policy roundtable meetings can be organised. Policy dialogues and roundtables are a potentially powerful mechanism for evidence-informed policy development and knowledge exchange that can help strengthen health systems, governance and sustainability. While there is keen interest in centralised intake for scheduled clinical services from across Manitoba, further use and evaluation of SEMs is still required. A deliberative policy dialogue process holds promise for creating an environment that enables all relevant voices to be heard, and for effective, evidence-informed policy to be developed.

## Abbreviations

SEM: Single-entry model
TJR: Total joint replacement surgery of the hip or knee
WCIS: Winnipeg Central Intake Service
WRHA: Winnipeg Regional Health Authority

## References

[1] Abelson J, Forest P-G, Eyles J, Smith P, Martin E, Gauvin F-P (2003). Deliberations about deliberative methods: issues in the design and evaluation of public participation process. Soc Sci Med.

[2] Walt G, Shiffman J, Schneider H, Murray SF, Brugha R, Gilson L (2008). “Doing” health policy analysis: methodological and conceptual reflections and challenges. Health Policy Plan.

[3] Kingdon J (1984). Agendas, alternatives and public policies.

[4] Inglehart R (1995). Changing values, economic development and political change. Int Sci J.

[5] Innes JE, Booher DE. Collaborative dialogue as a policy making strategy. Berkeley: Institute of Urban and Regional Development; 2000.

[6] Gauvin F-P. Evaluating deliberative processes. Contract No.: 1202. Quebec: National Collaborating Centre for Healthy Public Policy; 2010.

[7] Lavis JN, Boyko JA, Gauvin F-P. Evaluating deliberative dialogues focussed on healthy public policy. BMC Public Health. 2014;14:1287. 10.1186/1471-2458-14-1287 PMC4301941 25516355

[8] Boyko JA, Lavis JN, Abelson J, Dobbins M, Carter N (2012). Deliberative dialogues as a mechanism for knowledge translation and exchange in health systems decision-making. Soc Sci Med.

[9] Rajan D, Adam T, Husseiny DE, Porignon D, Ghaffar A, Schmets G (2015). Policy dialogue: What it is and how it can contribute to evidence-informed decision-making.

[10] Grimshaw J, Eccles M, Lavis J, Hill S, Squires J (2012). Knowledge translation of research findings. Implement Sci.

[11] Lavis JN, Hammil A, Gildiner A, McDonagh R, Wilson M, Ross S (2005). A systematic review of the factors that influence the use of research evidence by public policymakers: Report submitted to the Canadian Population Health Initiative.

[12] Moat KA, Lavis JN (2013). 10 best resources for…evidence-informed health policy making. Health Policy Plan.

[13] Lavis J, Lomas J, Hamid M, Sewankambo N (2006). Assessing country-level efforts to link research to action. Bull World Health Organ.

[14] Wilson M, Lavis J, Grimshaw J (2012). Supporting the use of research evidence in the Canadian health sector. Healthcare Quart.

[15] Innvaer S, Vist G, Trommald M, Oxman A (2002). Health policy-makers’ perceptions of their use of evidence: a systematic review. J Health Serv Res Policy.

[16] Lavis J, Davies H, Oxman A, Denis J-L, Golden-Biddle K, Ferlie E (2005). Towards systematic reviews that inform health care and management and policy-making. J Health Serv Res Policy.

[17] Radcliffe M (2007). 18 weeks Ms Hewitt? I make it 14 months. Nurs Times.

[18] Canadian Institutes for Health Information (2015). Wait Times for Priority Procedures in Canada.

[19] Wait Time Alliance (2014). Time to Close the Gap: Report Card on Wait Times in Canada.

[20] Abelson J (2009). Opportunities and challenges in the use of public deliberation to inform public health policies. Am J Bioeth.

[21] Scutchfield F, Hall L, Ireson C (2006). The public and public health organizations: issues for community engagement in public health. Health Policy.

[22] OECD (2013). Waiting Time Policies in the Health Sector. OECD Health Policy Studies.

[23] Wait Times for Priority Procedures in Canada, 2014. Canadian Institute for Health Information. 2014. https://secure.cihi.ca/free_products/2014_WaitTimesAiB_EN.pdf. Accessed 20 Sept 2015.

[24] Bates M, Robinson N, McComb J (2014). Direct admission to a pacing centre of patients who present urgently for pacing: prospective pilot study of feasibility and length of stay. Heart.

[25] Damani Z, Bohm E, MacKean G, Quan H, Noseworthy T, Marshall D (2014). A comprehensive case study of an orthopaedic surgery referral service in the Winnipeg Regional Health Authority: A single-entry model to manage waiting times for total joint replacement surgery of the hip and knee. Clin Invest Med.

[26] Damani Z, Bohm E, DeMone B, Wright B, Marshall D. Improving Wait Times: Single-entry models for hip and knee replacement patients. Canadian Institutes of Health Research Institute of Health Services and Policy Research (IHSPR). Policy Rounds Webinar. CIHR; 2014.

[27] Damani Z, Bohm E, MacKean G, Quan H, Noseworthy T, Marshall D. Findings from a post-policy implementation review of the Winnipeg Central Intake Service (WCIS): a single-entry model to manage referrals and waiting times for hip and knee replacement. Winnipeg: CIHR Healthcare Renewal Policy Analysis - Policy Roundtable Meeting; 2014.

[28] Damani Z, Bohm E, MacKean G, Quan H, Noseworthy T, Marshall D. Comprehensively evaluating a single-entry model for elective surgery in Winnipeg: a multi-stakeholder national project under the CIHR Evidence-Informed Healthcare Renewal (EIHR) Roadmap Signature Initiative. Calgary: 1st Annual McCaig Meeting on Osteoarthritis and Musculoskeletal Diseases; 2014.

[29] Damani Z, Bohm E, Conner-Spady B, MacKean G, Quan H, Noseworthy T, et al. Single-Entry Models: The Patient Perspective Taming of the Queue Pre-Conference Workshop. Ottawa; 2014.

[30] Morestin F, Gauvin F-P, Hogue M-C, Benoit F (2010). Method for synthesizing knowledge about public policies.

[31] Abelson J, Montesanti S, Li K, Gauvin F-P, Martin E (2010). Effective strategies for public engagement in the development of healthcare policies and programs.

[32] CIHR. Roadmap Signature Initiative in Evidence-Informed Healthcare Renewal: Healthcare Renewal Policy Analysis: Canadian Institutes for Health Research. 2012. https://www.researchnet-recherchenet.ca/rnr16/vwOpprtntyDtls.do?prog=1568&view=currentOpps&org=CIHR&type=AND&resultCount=25&sort=program&all=1&masterList=true. Accessed 20 Sept 2015.

[33] Lavis JN, Boyko JA, Oxman AD, Lewin S, Fretheim A (2009). SUPPORT Tools for evidence-informed health Policymaking (STP) 14: Organising and using policy dialogues to support evidence-informed policymaking. Health Res Policy Syst.

[34] Lomas J, Culyer T, McCutcheon C, McAuley L, Law S (2005). Conceptualizing and combining evidence for health system guidance.

[35] Hsieh H, Shannon SE (2005). Three Approaches to Qualitative Content Analysis. Qual Health Res.

[36] Multi-stakeholder summit (2011). The referral and consultation process: making the system work better for patient outcomes.

[37] Buse K, Mays N (2005). Making health policy.

